# The Malignancy Potential of Porokeratosis: A Single-Center Retrospective Study

**DOI:** 10.7759/cureus.13083

**Published:** 2021-02-02

**Authors:** Taylor Novice, Mio Nakamura, Yolanda Helfrich

**Affiliations:** 1 Dermatology, University of Michigan, Ann Arbor, USA

**Keywords:** malignant transformation, non-melanoma skin cancer, squamous cell carcinoma, basal cell carcinoma, melanoma, porokeratosis

## Abstract

Background

Porokeratosis (PK) is a rare group of keratinization disorders. While the overall prognosis of PK is favorable, malignant transformation of PK to skin cancer has been reported in 6.9% to 11.6% of the cases. Prior estimates of malignant transformation of PK have been based on reviews of published cases, which introduces possible publication bias. We aim to eliminate this potential bias and quantify the characteristics, risk factors, and malignancy potential of PK.

Methodology

A single-center retrospective chart review of patients with a diagnosis of PK was conducted.

Results

In this study, 6.4% to 16.4% of histologically confirmed PK lesions demonstrated malignant transformation. A higher proportion of disseminated superficial actinic porokeratosis (DSAP) cases (as high as 29.3%) showed malignant transformation compared to PK of Mibelli (as high as 6.0%). Out of the two cases of linear PK, both demonstrated malignant transformation.

Conclusions

In summary, PKs are at risk for malignant transformation, and patients with DSAP and linear PK, in particular, should receive more long-term surveillance. Limitations of this study include the inability to control for confounding factors due to the retrospective nature and the small size of our cohort.

## Introduction

Porokeratosis (PK) is a rare group of keratinization disorders first described by Vittorio Mibelli in 1889, presenting as annular lesions with an atrophic center and a keratotic ridge [1,2]. Common subtypes of PK include porokeratosis of Mibelli (PM), disseminated superficial actinic porokeratosis (DSAP), PK palmaris, plantaris, et disseminata (PPPD), punctate PK, and linear PK [2]. Although the exact pathophysiology is unknown, PK is thought to represent a focal, expanding clone of abnormal keratinocytes [3-5].

While the overall prognosis of PK is favorable, it has been postulated to be a premalignant condition [6-9]. Otsuka et al. found that epidermal cells of PK demonstrate DNA ploidy indicating a neoplastic process [6]. Cultured fibroblasts derived from the affected skin also show chromosomal abnormality and a clonal population of cytogenetically abnormal cells [7]. Chromosomal instability and high frequency of chromosomal aberration have been shown in the skin of patients with PK following X-ray irradiation [9,10]. Malignant transformation of PK to squamous cell carcinoma (SCC) and Bowen’s disease (SCC in situ) have been reported in 6.9% to 11.6% of cases [8,11-14].

These prior estimates of malignant transformation of PK have been based on reviews of published cases, which introduces possible publication bias. We aim to eliminate this potential bias and quantify the characteristics, risk factors, and malignancy potential of PK through a retrospective review of patients with PK presenting to our institution.

## Materials and methods

A retrospective medical chart review of patients who had a diagnosis of PK in the Michigan Medicine Department of Dermatology was conducted. DataDirect, a data query system, was used to identify patients from 2000 to 2017 who had International Classification of Diseases (ICD)-9 or 10 diagnostic codes for PK. EMERSE, an electronic medical search engine, was used to confirm the diagnosis of PK, as some of the ICD-9 and 10 codes for PK included other diagnoses. EMERSE was then used to search the notes to identify patients who had a diagnosis of both PK and skin cancer.

The medical records of all identified patients with PK were reviewed for information on demographics and characteristics of PK. Medical records were also reviewed for histologic confirmation of PK as well as the number and location(s) of skin cancer(s). Potential risk factors for PK including immunosuppression status or immunosuppressive medications, radiation, and UV therapy and their duration were also recorded. Family history or genetic predisposition to PK were also documented.

We used clinical notes and histopathological evidence to determine if a PK transformed into a skin cancer. Only histologically confirmed cases of PK were included. Due to the different levels of certainty in whether malignant transformation had occurred, malignant transformation was defined under three different categories. Definitive malignant transformation was defined by either (1) clinical or photographic documentation that a PK transformed into a skin cancer with confirmatory pathologic diagnosis or (2) histologic findings of malignant transformation. Probable malignant transformation was defined by a PK and histologically proven skin cancer occurring in the same location (i.e., “left lateral thigh” as the location for both PK and skin cancer). Possible malignant transformation was defined as PK and skin cancer occurring in the same general anatomic location (i.e., “left lower extremity”).

Medical records were reviewed and data were collected separately by two investigators and reviewed for consistency. Inconsistencies were resolved by discussion between the two investigators. This study was deemed exempt by the University of Michigan Institutional Review Board.

Data were summarized as means and standard deviations (SDs) for continuous variables and frequencies and percentages for categorical variables. Unadjusted odds ratios (ORs) and 95% confidence intervals (CIs) were calculated to compare risk factors between patients who did and did not experience malignant transformation. Variables with unadjusted ORs with P < 0.05 were included in a multivariable logistic regression model. Statistical significance was defined as P < 0.05. Statistical analyses were conducted using SAS version 9.4 (SAS Institute Inc., Cary, NC, USA).

## Results

Patient selection

**Figure 1:**
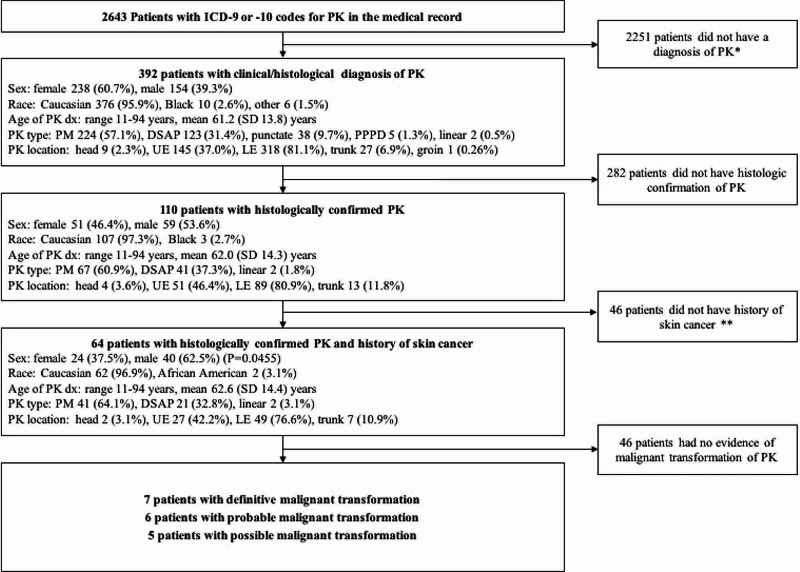
Flowchart of the patient selection process and patients’ demographic/clinical characteristics. DSAP, disseminated superficial actinic porokeratosis; dx, diagnosis; LE, lower extremity; PK, porokeratosis; PM, porokeratosis of Mibelli; PPPD, porokeratosis palmaris, plantaris, et disseminata; UE, upper extremity * search terms used at this stage include “porokeratosis,” “disseminated superficial porokeratosis,” and “DSAP” ** search terms used at this stage include “skin cancer,” “NMSC,” “basal cell,” “squamous cell,” “Bowen,” “melanoma,” “MIS,” “BCC,” and “SCC.” The diagnosis of skin cancer was confirmed by reviewing histopathology reports

Figure 1 shows the flowchart of the patient selection process and the patients’ demographic and clinical characteristics. A total of 2,643 patients were identified with ICD-9 or 10 codes that could include PK. On further review of these codes and elimination of other non-PK conditions, only 392 patients had a diagnosis of PK in the medical record and 110 were found to have histological confirmation of PK. A total of 64 of these patients also had a history of skin cancer. Of these 64 patients, seven had definitive malignant transformation of their PK, six had probable malignant transformation, and five had possible malignant transformation; the other 46 had skin cancers with no apparent connection to PK.

Characteristics of patients with histologically confirmed diagnosis of PK

The characteristics of the 392 patients with a clinical diagnosis of PK and the 110 patients with histological confirmation of PK are shown in Figure 1. Of the 110 patients with histologically confirmed PK, a total of 64 patients also had a history of skin cancer either before or after the PK diagnosis. The characteristics of these 64 patients were similar to all patients with histologically confirmed PK, as described above (Figure 1). The details of the number and types of skin cancers before and after the PK diagnosis is shown in Table 1. Note that the total number of skin cancers is greater than 64 because some patients had multiple cancers of varying subtypes and some patients had cancer both before and after the diagnosis of PK. Treatment for PK was provided to approximately one-third of patients (18 [28.1%]) with the most common treatments being topical fluorouracil and cryotherapy. Table 2 breaks down patient characteristics and exposure history by type of PK.

**Table 1:** Number of patients with previous and subsequent skin cancers among the 110 patients with histologically confirmed PK. BCC, basal cell carcinoma; MIS, PK, porokeratosis; melanoma in situ; SCC, squamous cell carcinoma; SCCIS, squamous cell carcinoma in situ * Other skin cancers included one patient with a previous keratoacanthoma, one patient with a subsequent keratoacanthoma and a subsequent eccrine carcinoma, and one patient with a subsequent vulvar intraepithelial neoplasia

Previous skin cancers								
Number of skin cancers	SCC/SCCIS		BCC		Melanoma/MIS		Other*	
	N	%	N	%	N	%	N	%
0	39	60.9	30	46.9	52	81.3	63	98.4
1-2	18	28.1	23	35.9	12	18.8	1	1.6
3-4	2	3.1	4	6.3	0	0	0	0
5-9	3	4.7	6	9.4	0	0	0	0
10+	2	3.1	1	1.6	0	0	0	0
Subsequent skin cancers								
Number of skin cancer	SCC/SCCIS		BCC		Melanoma/MIS		Other*	
	N	%	N	%	N	%	N	%
0	35	54.7	32	50.0	60	93.8	62	96.9
1-2	15	23.4	20	31.3	4	6.3	2	3.1
3-4	5	7.8	3	4.7	0	0	0	0
5-9	6	9.4	7	10.9	0	0	0	0
10+	3	4.7	2	3.1	0	0	0	0

**Table 2:** Demographic and clinical characteristics of the 64 patients with histologically confirmed diagnosis of PK and a history of skin cancer by type of PK. DSAP, disseminated superficial actinic porokeratosis; H/N, head/neck; LE, lower extremity; PK, porokeratosis; PM, porokeratosis of Mibelli; SD, standard deviation; UE, upper extremity; UV Tx, ultraviolet therapy; yrs, years * Percentages do not necessarily add up to 100% because some patients had PK lesions on multiple locations

Type	N	Male: female N:N	Age of diagnosis Mean (SD) yrs	Location N (%)*	Immunosuppression N (%)	Radiation exposure N (%)	UV Tx exposure N (%)
PM	41	30:11	66.5 (11.4)	H/N: 2 (4.9%) UE: 9 (22.0%) LE: 30 (73.2%) Trunk: 4 (9.8%)	16 (39.0%)	7 (17.1%)	1 (2.4%)
DSAP	21	10:11	58.1 (14.1)	H/N: 0 (0%) UE: 16 (76.2%) LE: 18 (85.7%) Trunk: 3 (14.3%)	6 (28.6%)	3 (14.3%)	1 (4.8%)
Linear	2	0:2	30.5 (27.6)	H/N: 0 (0%) UE: 2 (100%) LE: 1 (50%) Trunk: 0 (0%)	1 (50%)	1 (50%)	0 (0%)
Total	64	40:24	62.6 (14.4)	H/N: 2 (3.1%) UE: 27 (42.2%) LE: 49 (76.6%) Trunk: 7 (10.9%)	23 (35.9%)	11 (17.2%)	2 (3.1%)

Malignant transformation of histologically confirmed PK

Of the 110 patients with histologically confirmed PK, seven had definitive evidence of malignant transformation of their PK (6.4%), six had probable malignant transformation (5.4%), and five had possible malignant transformation (4.5%). The details of each of the definitive cases are presented in Table 3.

**Table 3:** Details of the seven cases of definitive malignant transformation of PK into skin cancer. DSAP, disseminated superficial actinic porokeratosis; dx, diagnosis; LE, lower extremity; MT, malignant transformation; PK, porokeratosis; PM, porokeratosis of Mibelli; SCC, squamous cell carcinoma; SCCIS, squamous cell carcinoma in situ; UE, upper extremity; yrs, years * Patient has had porokeratoses for years, but it is not clear in the notes when exactly it started ** The note clearly stated that a prior DSAP lesion had changed and biopsy was obtained, which showed malignancy

Porokeratosis			Malignant Transformation							
Age of dx (yrs)	Type	Type of treatment	Age of dx (yrs)	Duration of PK at time of MT (yrs)	Location	Type of skin cancer	Evidence of MT	Number of other skin cancers	Immune suppression	Radiation therapy
73	DSAP	None	77	4	LE	SCCIS	Histologic confirmation	10	no	no
11	Linear	Cryotherapy, tazarotene	62	51	LE	SCC	Histologic confirmation	1	no	no
71	PM	None	75	4	LE	SCC	By photograph and clinical description**	1	no	no
50	Linear	None	62	12	UE	SCC	By clinical description**	4	yes	yes
41	DSAP	Tretinoin 0.05%	41	0*	LE	SCCIS	Histologic confirmation	3	no	no
72	PM	Silvadene cream and clobetasol	85	13	LE	SCC	By clinical description**	2	yes	no
55	DSAP	5-fluorouracil	51	0*	UE	SCCIS	By clinical description**	14	yes	yes

Risk factors for malignant transformation of histologically confirmed PK

Of the patients with histologically confirmed PK, the demographic and clinical characteristics of patients who had malignant transformation of their PK and those who did not were compared (Table 4). All patients with definitive, probable, or possible malignant transformation were included in this analysis. Overall, PK was diagnosed at an earlier age in patients who had malignant transformation, but this finding was not statistically significant (56.4 [SD: 18.7] vs. 63.1 [SD: 13.1], p = 0.333, not shown in Table 4). PM was less likely to transform into skin cancer compared to DSAP (OR: 0.153, 95% CI: 0.046-0.517, P = 0.0016). Subsequent melanoma and other skin cancers were also associated with a decreased risk of malignant transformation of PK (melanoma: OR: 0.054, 95% CI: 0.007-0.422, P = 0.0002; other skin cancer: OR: 0.059, 95% CI: 0.008-0.461, P = 0.0004). All other factors such as sex, race, PK location, and exposure history were similar between the two groups (Table 4). After adjusting for all statistically significant variables, only type of PK (DSAP more likely than PM) was a significant predictor for malignant transformation (P = 0.0012) (Table 4). Family history or genetic predisposition to PKs was not clearly recorded in patient records and were not analyzed.

**Table 4:** Comparison of demographic and clinical characteristics of patients who had malignant transformation of their PK and those who did not among the histologically confirmed cases (includes definitive, probable, and possible cases of malignant transformation). BCC, basal cell carcinoma; CI, confidence interval; DSAP, disseminated superficial actinic porokeratosis; MIS, melanoma in situ; NS, not specified; OR, odds ratio; PK, porokeratosis; PM, porokeratosis of Mibelli; SCC, squamous cell carcinoma; SCCIS, squamous cell carcinoma in situ; UV, ultraviolet * Statistically significant (p < 0.05)

		Malignant transformation		Unadjusted			Adjusted			
		Yes (N)	No (N)	OR	95% CI	P-Value	OR		95% CI	P-Value
Demographic information										
Gender	Female	12	39	2.718	0.938-7.874	0.0730	--	--		--
	Male	6	53							
Race	African American	1	2	2.647	0.227-30.853	0.4181	--	--		--
	Caucasian	17	90							
PK characteristics										
PK type	PM	4	63	0.153	0.046-0.517	0.0016*	0.101	0.025-0.406		0.0012*
	DSAP	12	29							
	Linear PK	2	0	--	--	--	--	--		--
Treatment	No	12	65	0.831	0.283-2.441	0.7813	--	--		--
	Yes	6	27							
PK location										
Head/Neck	Involved	0	4	--	--	--	--	--		--
	Uninvolved	18	88							
Upper extremity	Involved	11	40	2.043	0.727-5.742	0.2021	--	--		--
	Uninvolved	7	52							
Lower extremity	Involved	16	73	2.082	0.440-9.852	0.5156	--	--		--
	Uninvolved	2	19							
Trunk	Involved	2	11	0.921	0.186-4.555	1.00	--	--		--
	Uninvolved	16	81							
Skin cancer history										
Previous SCC/SCCIS	Yes	8	62	0.387	0.139-1.081	0.1057	--	--		--
	No	10	30							
Previous BCC	Yes	7	72	0.177	0.061-0.515	0.0015*	0.541	0.146-2.000		0.3568
	No	11	20							
Previous melanoma/MIS	Yes	3	54	0.141	0.038-0.520	0.0015*	0.379	0.060-2.400		0.3029
	No	15	38							
Previous other skin cancer	Yes	0	46	-	-	-	--	--		--
	No	18	46							
Subsequent SCC/SCCIS	Yes	11	63	0.723	0.255-2.056	0.5879	--	--		--
	No	7	29							
Subsequent BCC	Yes	11	66	0.619	0.217-1.770	0.4049	--	--		--
	No	7	26							
Subsequent melanoma/MIS	Yes	1	48	0.054	0.007-0.422	0.0002*	0.857	0.036-20.671		0.9242
	No	17	44							
Subsequent other skin cancer	Yes	1	46	0.059	0.008-0.461	0.0004*	0.057	0.002-1.372		0.0745
	No	17	46							
Exposure history										
Immune suppression	No	12	64	0.875	0.298-2.566	0.7869	--	--		--
	Yes	6	28							
UV therapy	No	17	91	0.187	0.011-3.133	0.3018	--	--		--
	Yes	1	1							
Radiation therapy	No	13	81	0.353	0.105-1.182	0.135	--	--		--
	Yes	5	11							

## Discussion

We analyzed 110 cases of histologically confirmed PKs and found that 6.4% to 16.4% of PK lesions demonstrated malignant transformation. Our malignant transformation rate of 6.4%-16.4% is similar to previously reported frequencies, which range 6.9%-11.6% [8,11-13]. Sasson and Krain reviewed 281 published cases of PK and found malignant transformation in 7.5% of the cases [11]. As these frequencies were determined based on review of published case reports, inflation due to publication bias was possible. We found a wider range of malignant transformation rates, possibly due to broader inclusion criteria for defining malignant transformation. Especially for DSAP, it was difficult to determine whether a PK truly transformed into a skin cancer or if the two lesions occurred in a similar location. We included probable and possible cases to provide a more conservative estimate of malignant transformation. We postulate that the true malignancy transformation rate falls within the middle of our range (6.4%-16.4%) and is likely to fall closer to previously published rates of 6.9%-11.6%.

Our study supports previous findings that SCC is the most common type of skin cancer to arise from a PK [11,15]. In our cohort, all cases of definitive malignant transformation were to SCC or SCCIS. When including probable malignant transformation, DSAP was shown to transform to BCC, consistent with previous studies showing that BCCs uncommonly arise within a lesion of PK [11,16,17]. Interestingly, we also noted one probable case of melanoma arising in association with DSAP. In a small retrospective chart review of 11 patients with PK, Maubec et al. found the first case of melanoma associated with PK [16]. They postulated that PK may be a premalignant lesion for melanoma; however, it is possible that these two lesions occurred incidentally in a similar location.

We found a significantly higher risk of malignant transformation in DSAP, which differs from previously published reports. Sasson and Krain found that linear PK and large PM lesions were more likely to transform into a skin cancer (19%), while smaller DSAP and punctate PK had a much lower risk (3.4% and 7.6%, respectively) [11]. While we did not evaluate PK size or number due to inconsistent availability of this information in medical records and there are limitations to accurately determining malignant transformation of DSAP through chart review, our results suggest the need for heightened surveillance of these lesions, as they have the potential for transformation to cutaneous malignancy. Our results support the previous findings that linear PK has a high risk of transforming into skin cancer; there were only two cases of histologically confirmed linear PK, but both cases transformed into SCC.

Overall, many patients with PK had a history of at least one non-melanoma skin cancer before or after the diagnosis of PK. However, a positive skin cancer history before or after PK diagnosis was not associated with an increased risk of malignant transformation of a PK. To our knowledge, this is the first study that investigated whether history of skin cancer was a risk factor for transformation of PK. Our findings support other studies that have suggested that intrinsic qualities of a PK lesion make it more likely to transform rather than traditional risk factors for skin cancers, including immunosuppression and cumulative UV exposure [6-10,15]. We also found that immunosuppression or history of radiotherapy or UV therapy were not risk factors for malignant transformation of PK, which is supported by the findings of Sasson and Krain [11]. Furthermore, in our cohort, location of the PK was not a significant risk factor for malignant transformation. We were not able to determine risk of malignant transformation based on prior treatment of PK given the limited sample size.

Due to the retrospective nature of this study, limitations of our study include lack of control for confounding factors including prior UV exposure, which is implicated in the development of both PK and skin cancer. We used histologic confirmation of PK rather than relying on clinical diagnosis alone to limit potential errors regarding the accuracy of information and physician variability in diagnosis; however, we acknowledge that this decision may limit our findings as more atypical-appearing PK concerning for a skin cancer may be more likely to be biopsied than classic-appearing PK. Identification of malignant transformation in a retrospective study can prove challenging; however, we relied on histologic evidence and/or clinical documentation of a PK transforming into skin cancer to minimize this limitation. In our cohort, malignant transformation occurred up to 51 years from the diagnosis of the PK; therefore, it is likely that some patients who may have had malignant transformation were lost to follow-up and were not captured in our data. Last, we acknowledge the small sample size of our study and the consequent limitations in the multivariable analysis. However, this study is the largest retrospective study to our knowledge that assesses the carcinogenic risk of PK. Large-scale prospective studies that follow PK patients long-term for the development of skin cancer are needed to corroborate our findings.

## Conclusions

A retrospective chart review of 110 patients with histologically confirmed PK found that 6.4%-16.4% of the cases of PK transformed into a skin cancer. DSAP was more likely to show transformation into skin cancer compared to PM. Exposures including immunosuppression, radiotherapy, UV therapy, and a personal history of skin cancer were not associated with increased risk of malignant transformation. Although the etiology of malignant transformation remains unclear, PKs are at risk for malignant transformation, and patients with DSAP and linear PK, in particular, should receive more long-term surveillance.
